# A Prediction Model of Autism Spectrum Diagnosis from Well-Baby Electronic Data Using Machine Learning

**DOI:** 10.3390/children11040429

**Published:** 2024-04-03

**Authors:** Ayelet Ben-Sasson, Joshua Guedalia, Liat Nativ, Keren Ilan, Meirav Shaham, Lidia V. Gabis

**Affiliations:** 1 Department of Occupational Therapy, Faculty of Social Welfare and Health Sciences, University of Haifa, Haifa 3498838, Israelliatnativ@gmail.com (L.N.);; 2 Maccabi Healthcare Services, Tel-Aviv 6812509, Israel; gabis_l@mac.org.il; 3 Pediatrics, Faculty of Medicine and Health Sciences, Tel-Aviv University, Tel-Aviv 6997801, Israel; 4 Keshet Autism Center Maccabi Wolfson, Holon 5822007, Israel

**Keywords:** autism spectrum disorders, development, screening, machine learning, electronic health records

## Abstract

Early detection of autism spectrum disorder (ASD) is crucial for timely intervention, yet diagnosis typically occurs after age three. This study aimed to develop a machine learning model to predict ASD diagnosis using infants’ electronic health records obtained through a national screening program and evaluate its accuracy. A retrospective cohort study analyzed health records of 780,610 children, including 1163 with ASD diagnoses. Data encompassed birth parameters, growth metrics, developmental milestones, and familial and post-natal variables from routine wellness visits within the first two years. Using a gradient boosting model with 3-fold cross-validation, 100 parameters predicted ASD diagnosis with an average area under the ROC curve of 0.86 (SD < 0.002). Feature importance was quantified using the Shapley Additive explanation tool. The model identified a high-risk group with a 4.3-fold higher ASD incidence (0.006) compared to the cohort (0.001). Key predictors included failing six milestones in language, social, and fine motor domains during the second year, male gender, parental developmental concerns, non-nursing, older maternal age, lower gestational age, and atypical growth percentiles. Machine learning algorithms capitalizing on preventative care electronic health records can facilitate ASD screening considering complex relations between familial and birth factors, post-natal growth, developmental parameters, and parent concern.

## 1. Introduction

Autism spectrum disorders (ASD) manifest through social communication deficits and atypical behaviors, as listed in The Diagnostic and Statistical Manual of Mental Disorders, Fifth Edition (DSM-5) [1] and it may affect 1:36 individuals [2]. Nonetheless, the prevalence is highly variable between countries, with lower prevalence in lower income communities [3,4], depending primarily on the rate of detection and diagnosis. Recent rates of diagnosis in Israel are 1% among 2–3-year-olds and 2% in 4–5-year-olds [5], which is close to the global median of 1% [4] and higher than previous reports [3]. Early intervention administered before three years of age leads to significantly greater social communication, adaptive functioning, and cognitive competencies [6,7]. The American Academy of Pediatrics and other health organizations recommend [8,9,10] early screening for ASD at 18 and 24 months during dedicated well-baby visits to promote the initiation of early intervention and improve outcomes [11,12,13]. In Israel, the well-baby check-ups are performed at a national level and regulated by the Ministry of Health (MoH). The age distribution of developmental milestones examined during the scheduled well-baby visits has been recently studied in this national database [14,15]. The current study focused on a model detecting children with elevated ASD markers in routine well-baby check-up records to provide higher accuracy of ASD detection during developmental screening. Such a model can, in the long run, be implemented to alert healthcare providers during routine checkups to follow-up with ASD-specific screening tools.

Behavioral markers linked to ASD can be observed during the first and second years of life, even in low-risk populations (e.g., [7,16,17,18,19]), yet the average age of diagnosis worldwide remains above three years of age [20,21]. One of the barriers to early detection of ASD is the great heterogeneity in the ‘red flags’, onset, and progression of symptoms, and in the divergence of developmental trajectories in ASD [18,22]. Early detection of ASD relies upon behavioral observations in the social communication and sensory motor domains (e.g., [7,16,23]), and on observing developmental trajectories [7]. Deviations in motor and social communication development significantly differentiated the ASD group from typically developing children and children with other delays during the first [22,24] and second year of life [18,25]. Some early ASD signs can be easily missed by a healthcare provider during a short checkup visit due to their gradual deviation or subtlety, such as social reciprocity, social smile, and eye contact [11,22]. Furthermore, disparities in healthcare screening resources and in provider awareness of individual developmental differences [7,26] challenge early detection. To facilitate and adapt the screening level to different trajectories, the screening procedure needs to match the risk level of the child [27]. Including known risk factors and individual differences may highlight specific variations and increase the level of awareness in particular populations, such as those with prematurity or familial factors [28,29,30,31].

Prematurity, like other factors, interacts with additional ASD risk factors, such as cesarean delivery, parental age, and birth complications; therefore, there is a need for an integrative/cumulative approach to risk estimation. These non-linear relationships can be detected using machine learning (ML) analysis of big data, as conducted in the present study. Research shows the value of ML model risk score predictions for accurately stratifying clinical populations [32] and can guide the provider to personalize the checkup according to their risk level. In the ASD literature, ML has been applied for various purposes, including differentiating subgroups [33] and, specifically, parsing the behavioral phenotype variance [34]. Deep learning methods have been applied for ASD detection based on motor abnormalities [35] and the prediction of behavioral intervention efficacy from patient data [36]. Previous electronic health record (EHR) data analysis by ML algorithms differentiated clusters of ASD patients with common co-morbidities (e.g., [37,38,39]). Furthermore, ML tools can predict ASD likelihood from family medical history in EHRs [40,41]. Such tools address the need for a cost efficient and accurate process which considers neurodevelopmental heterogeneity among children [42].

The well-baby clinic system in Israel provides nation-wide developmental checkups from birth to six years and offers an opportunity for implementing universal ASD screening [43]. The database was digitized to build and test a ML model that can identify significantly deviant early milestones linked to ASD from a child’s EHR at early stages of development and include specific background information, such as pregnancy and familial history. In the current study, we aimed to identify the most significant features linked to a subsequent diagnosis of ASD from a large database of EHRs using ML tools. The ML prediction of ASD was also verified by examining the proportion of children that were flagged as false positives by the model but manifested significant developmental delays. Since the analysis is driven by a large and diverse population, it may provide an applicable model for developmental surveillance and improve ASD detection below the age of two years.

## 2. Materials and Methods

### 2.1. Study Design and Ethics

This was a health records retrospective cohort study. This study was approved by the MoH Helsinki (#15/2021) committee. The need for consent was waived by the ethics committee. EHR data were recorded by the providers of the Israeli national well-baby developmental surveillance program called Tipat Halav (TH, “Drop of Milk”) managed by the MoH. Data were extracted and anonymized by the TIMNA project personnel (Israel Ministry of Health’s Big Data Platform). Data were extracted until 31 December 2021. Researchers accessed de-identified participants’ data through this dedicated secured MoH platform.

### 2.2. Data Source

The EHRs analyzed in this study reflected a cohort followed by the national well-baby MoH clinics. The MoH is the main provider of developmental checkups at 503 clinics across the country, covering 75% of the Israeli population. The remaining 25% are followed by two Health Maintenance Organizations according to the same national guidelines. The services include immunizations, growth, and developmental surveillance, as well as medical and environmental risk detection for the mother and child. The services are provided by nurses with specific training in maternal and child health and a developmental pediatrician. There are approximately 11 visits from birth to age 6 years, each with a set of actions based on the child’s age and risk status. The nurses enter the data in a digital EHR.

The developmental protocol of TH includes a checkup of progress in 60 age-related milestones across gross motor, fine motor, language, and personal–social developmental domains [43]. The milestones included in the TH protocol are based in part on the Denver Developmental Screening Test (DDST) [44]. At each of the nine age bins, from six weeks to six years, the nurses check between 4–9 milestones. There are 38 milestones tested up to 24 months. Performance results for each milestone are recorded. During developmental checkups, parents are prompted to report their concerns regarding the child’s development and/or hearing. An ASD-specific screening tool is not integrated into the checkups.

The EHR inclusion criteria were: (1) children born between 1 January 2014–31 December 2019; (2) the availability of at least two years of computerized data. This led to a database of 780,610 EHRs. In order to develop a model that minimizes data leakage from diagnosis to predictors, we included: (1) as predictors in the model measurements up to two years of age and (22) as the outcome metric of ASD, only children with an ASD diagnosis reported after two years of age. The average age at which diagnosis was recorded for the full ASD group was 37.5 months, SD = 13.3 months (13.22 months–7.72 years).

### 2.3. Case Definitions

The prediction of an ASD diagnosis was based on a diagnosis of ASD reported in the child’s EHR, either by an International statistical classification of diseases and related health problems (ICD-9 [45]) code of ASD or reports in provider notes after the age of two years. Following ICD-9 case identification, texts for the rest of the population were searched for ASD-related keywords and verified manually for indication of ASD in the child (as opposed to family members). Children with other conditions with the same acronyms as Pervasive Developmental Disorders (PDD) and ASD were excluded (e.g., Atrial Septal Defect: ASD). Of the total sample, 1275 children were labeled as ASD-diagnosed, 722 children were identified by their ICD-9 diagnostic code, and an additional 553 were identified from the clinical textual notes. In total, 1163 were diagnosed after the age of 2 years.

EHRs of the Typically Developing (TD) group were defined as records of children without an ASD or other pervasive developmental and medical conditions diagnosis. The TD group excluded EHR records of children with another non-ASD pervasive medical or developmental condition. Since there are multiple etiologies linked to ASD, such as Fragile X, Tuberous Sclerosis, metabolic disorders, and more than 200 single genetic disorders, we did not exclude specific etiologies except Down syndrome, as explained below. Global developmental delay and intellectual disability [46] can occur as a comorbidity of ASD or differential diagnosis and are defined as significant delay in more than one developmental domain with performance of at least two standard deviations below age norms. When reported as a comorbidity, ASD symptoms should prevail, according to DSM-5 ASD criteria—E (1). We excluded children who only had these diagnoses reported for them, since our goal was to build a system that can assist providers in noticing young children whom they routinely miss.

The only diagnosis that may be causative and linked to ASD but was excluded from our ASD analysis is Down syndrome. Although children with Down syndrome may also have ASD, we decided to exclude this diagnosis since this etiology is overtly obvious from birth and the routine checkups for infants with Down Syndrome are adapted to their different trajectories.

The rationale was to focus the model design on the prediction of ASD and eliminate the prediction of conditions that the provider/family are aware of very early on (e.g., Down syndrome). The list of ICD-9 diagnosis was reviewed by clinical experts, and a list of congenital or other conditions highly associated with developmental challenges was compiled to reach higher level of comparison norms in the control group. Based on this list, 4577 children were excluded from the TD group due to musculoskeletal and nervous system disorders (20.3%), chromosomal abnormalities (4.5%), hearing loss (3%), convulsive disorders (1.7%), Infantile Cerebral Palsy (0.8%), and Spina Bifida (0.6%).

### 2.4. Data Preparation

**Figure 1:**
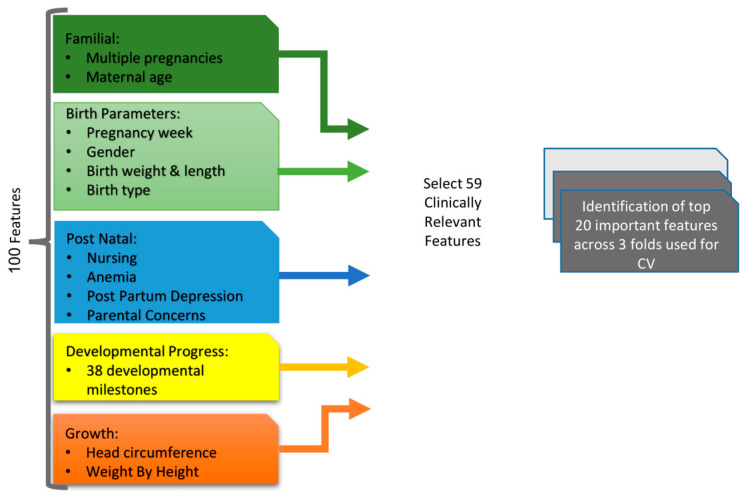
The iterative model design process from data mining to model testing.

The EHR structure and, consequently, the database, includes all documented information for a child and mother across all baby wellness visits. As such, the variables are a mix of structured (e.g., concern for development Yes/No, ICD-9 codes) and text data (e.g., provider notes), as well as fixed data points (e.g., birth weight) versus temporal data (e.g., head circumference measurements). For the structured features, there were continuous variables, such as birth weight in kg, and nominal variables, such as type of birth delivery (see Figure 1 for Model process).

As part of feature engineering, the distribution of variables in the database was investigated. Based on their distribution, several variables were recoded to enable their meaningful integration in the model. Most features included a considerable number of missing values (see Table 1, Table 2 and Table 3). Median imputation was applied in the case of continuous features, and a missing category was applied for categorical features. To address age differences in timing and number of visits, we calculated first and last measurements, median value, and standard deviation for longitudinal features. Each of the 38 developmental milestones entered the model as two features: only failed or ever passed. This configuration enabled the model to (1) account for cases in which a child had a milestone tested multiple times and (2) to differentiate not performing from a missing entry.

### 2.5. Analysis

#### 2.5.1. ML Modeling

Gradient boosting (CatBoost) method with a 3-fold cross validation was applied, a method implemented in EHR research [32,41]. Gradient boosting is a ML method that is a model evaluation technique where the dataset is divided into three subsets and the model is trained and tested three times, with two subsets for training and one for testing each time, to assess its performance across different data partitions. This method builds multiple decision trees, where each successive tree is fine-tuned to focus on the errors of the previous trees. The ensemble of trees is then used for the final prediction, working in a stage-wise manner rather than a parallel manner [47]. The high-risk group was defined as a predicted risk score above the Youden Threshold, an objective measure that sets a cutoff point that optimizes the sensitivity and specificity of the model [48]. The model was evaluated using AUC and its corresponding accuracy metrics. The model was designed by the team with the guidance of the third author, an expert in applying advanced machine learning techniques to predict outcomes from medical data.

We used the SHapley Additive exPlanations (SHAP) tool to quantify feature importance. The tool is employed to provide a comprehensive method for interpreting the results of ML models, attributing values to features to quantify their respective impacts on individual predictions. SHAP is a method used to explain the output of machine learning models by attributing the importance of each feature to the model’s predictions [49].

#### 2.5.2. Group Comparisons

The ASD and TD groups were compared across child and family characteristics, birth parameters, and familial and post-natal variables excluding missing values. For nominal variables, Chi square or Fisher’s exact tests were conducted with their corresponding measures of association, Cramer’s V, or Phi. Pairwise-adjusted Z-tests were used for variables with more than two categories. Normality tests showed that most continuous variables were normally distributed (*p* > 0.05), except for pregnancy week (*p* < 0.001). For normally distributed continuous variables, independent samples *t*-tests with Cohen’s d were conducted. Mann–Whitney U test compared pregnancy week between groups. A *p*-value threshold of 0.001 was applied given the large sample size [50] and multiple number of comparisons.

## 3. Results

### 3.1. Descriptive Statistics

The model analyzed EHRs of 774,778 TD children and 1163 ASD children (males 51.4%, 78.3% respectively). Table 1 presents the comparison of child and mother demographic features of TD versus ASD children. The TD group is equally distributed between birth years, while for the ASD group, as expected, there is a smaller proportion in 2019. There is a higher rate of ASD diagnosis for mothers born in Russia and Ethiopia. Table 2 presents familial and birth parameters, and Table 3 shows the post-natal features which were included in the training of our model. Tables show a significantly higher rate of preterm births in the ASD group, with older mothers, lower gestational week, lower birth weight, lower proportion of nursing, and much higher rates of parental concerns.

In the ASD group, there was an average of 13.7 visits overall (SD = 3.18; e.g., vaccination, growth, development) which was significantly (t (775,939) = −23.05, *p* < 0.001, d = 0.7) higher than for the TD group, with an average of 11.44 visits (SD = 3.26). Up to two years of age, the average age at the first visit was 0.85 months (SD = 1.37), and at the last visit, 18.94 months (SD = 2.85 months). The EHR visit ages indicated that, following the age of two years, the average age at the first visit was 28.19 months (SD = 0.26), and at the last visit, 37.37 months (SD = 13.93).

### 3.2. ML Model Predicting Risk Score for ASD

**Figure 2:**
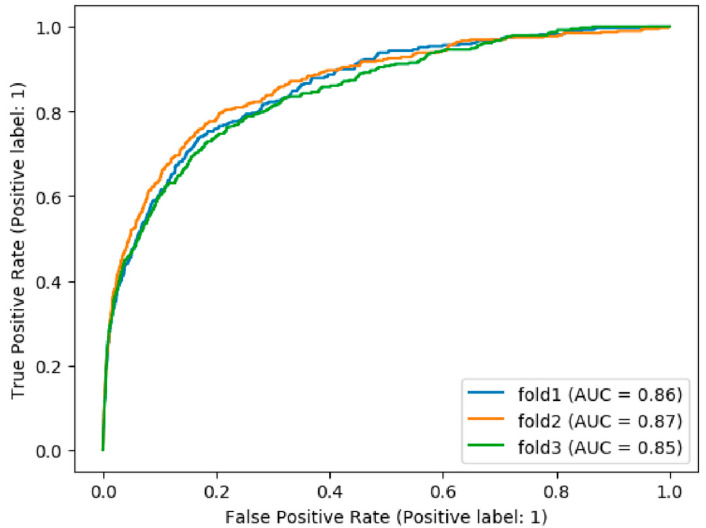
ROC curves for the 3 folds predicting ASD diagnosis.

The data initially included 100 features, from which we selected 59 clinically relevant features to build a 3-fold ML model (see Figure 1 and Table S1). All three folds showed similar results, with an average Area Under the ROC Curve (AUC) = 0.86 (SD < 0.009; see Figure 2) and corresponding sensitivity = 0.75 (SD = 0.03) and specificity = 0.81 (SD = 0.02). The average AUC for the training data was higher than the test (0.88, SD = 0.006), indicating that the model is slightly overfit to the training data, as expected (see Table S2 for AUC by fold). The full tested model classified a total of 81.4% children (n = 631,308) as low risk, and 18.6% children (n = 144,632) as high-risk. This high-risk group included 874 children with ASD, which is an incidence rate of 0.006, 4.3-fold higher than the ASD incidence in the entire cohort (0.001). We also present results for different distributions between sensitivity and specificity based on changing multiples of the Youden Threshold (see Table S3). Notable is the decrease in rates of false positive cases as threshold increases. In addition, comparing results from different methods showed overall stable AUC with a small variance between methods (see Table 4).

We reviewed the top 20 important features based upon their SHAP values within each fold. There was a high level of overlap between the top 20 features, with 15 (65%) in all three folds, 6 (25%) in two folds, and 3 (12.5%) unique to a specific fold, out of the total 24 unique features across the three folds (see Table S1).

### 3.3. Results from a Representative Fold

**Figure 3:**
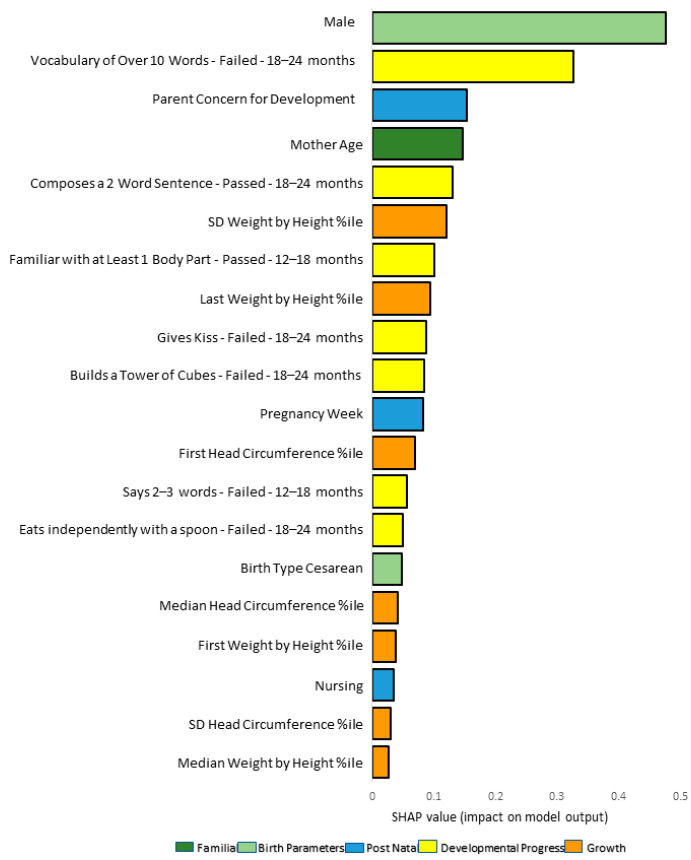
Feature importance bar plot for a representative fold from the full model. Note. The feature importance plot presents the importance of the features with their contribution to the model prediction as calculated using SHAP features. %ile: Percentile.

**Figure 4:**
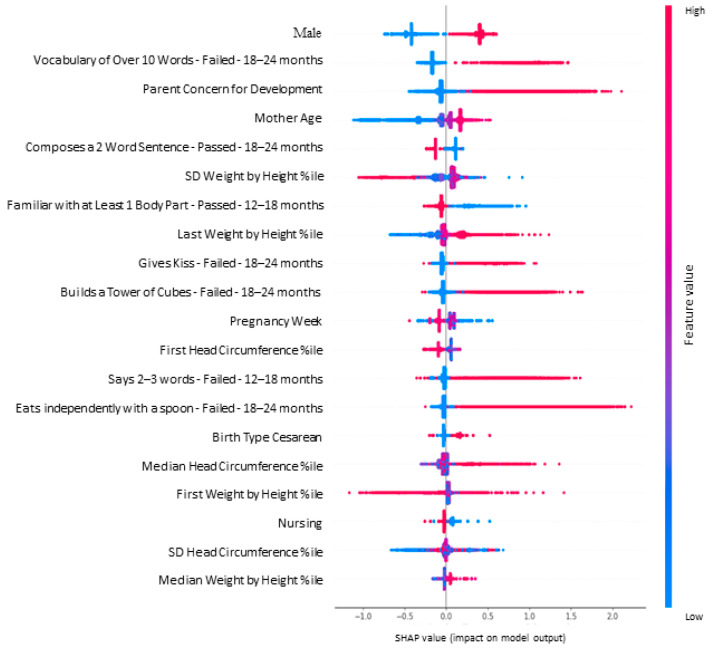
SHAP summary plot for a representative fold from the full model. Note. This plot represents the direction of the contribution of the individual values for each feature, to the right of the grey line indicates higher risk for ASD vs. to the left, indicating lower risk. The red reflects larger values, and the blue reflects lower values. %ile: Percentile.

**Figure 5:**
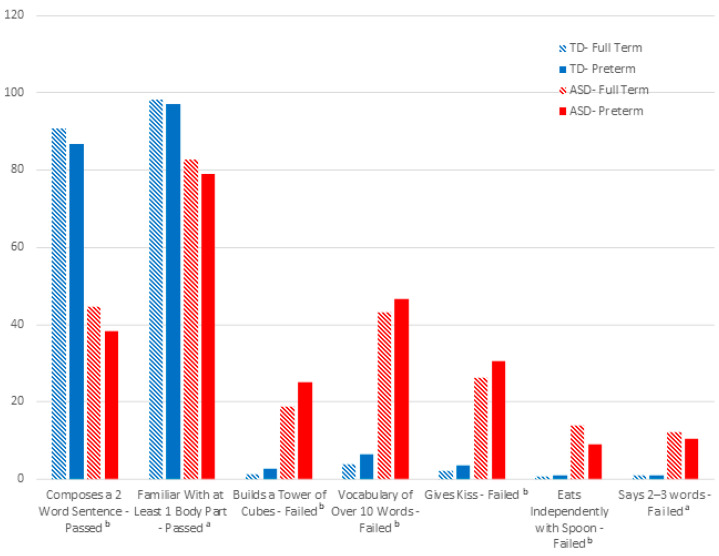
Comparison of percentages between groups considering prematurity for the seven important milestones consistent across folds. Note. One milestone entered as passed and the others as failed. All milestones in both ASD and TD showed no statistically significant difference between full term and preterm children. ^a^ 12–18 months. ^b^ 18–24 months.

We present a single fold as a representative model (see Figures S1 and S2 for the distribution of features in other folds). Model summary plots show the relative importance of 20 features and their distribution for a representative fold (see Figure 3 and Figure 4). Seven of the important features are related to developmental milestones from different domains. Fisher’s Exact tests showed significant differences in (*p* < 0.001) the rates of attaining seven important model milestones in the ASD group (see Figure 5):

1. has a vocabulary over 10 words (language domain), tested 18–24 months;
2. builds a tower from cubes (fine motor domain), tested 18–24 months;
3. gives kiss (personal–social domain), tested 18–24 months;
4. eats independently with spoon (personal–social domain), tested 18–24 months;
5. knows at least one body part (language domain), tested 12–18 months;
6. composes 2-word sentences (language domain), tested 18–24 months);
7. says 2–3 words (language domain), tested 12–18 months.

As can be seen in Figure 5, the group differences in the seven important milestones were not accounted for by prematurity; in other words, group comparisons were also significant when restricted to preterm versus full term samples (*p* < 0.001).

The remaining 13 features and their direction of association with ASD in this fold were as follows (8 of which overlap between folds. Features 10–13 appeared in the presented fold and not in all 3 folds):

1. boy
2. more parental developmental concerns
3. never nursing
4. advanced maternal age
5. lower pregnancy week (prematurity) (In some folds, pregnancy week was entered, and prematurity status in others)
6. lower weight by height percentile at first visit
7. higher weight by height percentile at last visit
8. larger weight by height percentile SD (larger variance across visits)
9. higher head circumference percentile at first visit
10. cesarian birth
11. higher head circumference percentile median (across visits)
12. larger head circumference percentile SD
13. higher weight by height percentile median (across visits)

### 3.4. Subgroup ML Analyses by Sex and Birth Year

Running a 3-fold cross validation model with boys only yielded a lower average AUC of 0.82 (SD = 0.03), with a sensitivity of 0.68 (SD = 0.02) and specificity of 0.82 (SD = 0.04). The average AUC for the training data (0.85, SD = 0.01) was slightly higher than the test (see Table S2 for AUC by fold). The 3 folds of the boys-only model had 12 overlapping features between folds out of the 29 unique features that appear in the 20 most important features in each fold. Of these, 10 features overlapped with the consistent features across the full model’s folds (see Table S4 and Figures S3–S5 for details of the boys-only model). Birth weight was a unique consistent feature for the boys-only model.

In addition, to verify stability of the results, we evaluated a model without children born in the year 2019 and saw stable results in the model’s AUC (see Table S4 for details of the model without 2019). The 2019-born children had a lower likelihood of ASD, therefore it was important to examine their impact on false positive results.

### 3.5. Validity of the Model’s High-Risk Group

To evaluate the high-risk status (i.e., false positive and true positive) identified by the above 3-fold model, we examined the rate of developmental provider referrals in the high risk versus low-risk groups (i.e., false negative and true negative). Provider’s developmental referral in the EHR was defined as a reported provider’s referral of the child for an extensive developmental assessment due to developmental delay. This referral variable was not included as a feature in the model and was therefore an external validity indicator of the model’s classification. Those who the model identified as high-risk had a 3.29 odds ratio (95% CI 3.18–3.39) of being referred for developmental delay than the low-risk group.

## 4. Discussion

This study demonstrates how ML models can utilize existing routine preventative care data to estimate a child’s ASD risk prior to the age of 2 years. The model predicting early ASD signs capitalized on the ongoing recording of real-life data by baby wellness providers, leading to a rich array of contributing real-world clinical measurements, including biometric predictors, such as familial, birth, post-natal, growth, and clinical predictors of developmental progress. At the healthcare policy level, this evidence calls for integrating technology to facilitate ASD detection in routine care. This is crucial for earlier detection of ASD globally, overcoming screening barriers related to access to high quality healthcare. The model detected 75% of ASD children and remained relatively stable in performance when only boys were included. Such a system can flag risk factors in a child’s early profile and facilitate a formal ASD screening during routine checkups [29]. While the model tested was slightly overfit to training data, it displayed similar results. Furthermore, this study presents the impact of different thresholds upon the balance of sensitivity and specificity. As threshold increases, the number of false positive cases decreases at the cost of fewer true positives. Healthcare providers can select different thresholds to support their context-specific policy and decision making. Future research with ample data would enable employment of methods such as CatBoost overfit detection during the training process to prevent overfitting. The current model’s contributing factors represented well-known features—being a boy, lower pregnancy week, older mother’s age, parental developmental concern, delayed language and personal–social development [51,52]—and less known features—never nursing [53], fine motor delay [54], and higher growth percentiles [55]. While the latter have some supporting evidence, they are not well-established factors. The model highlighted the most significant atypical developmental features among those that are well-known. Such discoveries are made possible by ML algorithms learning the interactions between features and improving the power of features by specific combination of familial data and examination of the child. The early ASD diagnosis process must start with identifying a child’s biometric and clinical data. Integrating ML tools as decision support systems in EHRs offers a timely efficient way to alert providers regarding a young child’s ASD risk.

Of the 38 milestones checked up to 24 months, six were important features consistent across the model folds. These milestones reflect a mixture of the language (three milestones), personal–social (two milestones) and fine motor (one) domains. Most were from the 18–24 months checkup, but one was earlier, from 12–18 months (i.e., knows at least one body part). While language expression and comprehension delay are not a diagnostic feature of ASD, they appear to reflect the child’s social communication challenges early on and are easier for providers and parents to observe. This is consistent with evidence of robust divergence in ASD from 14–24 months, specifically in language and social development [18,22,24]. These important milestones were identified by the model via their interactions among themselves and with other features. Nonetheless, differences between ASD and TD groups on each of the six milestones were not explained by prematurity or by sex differences, indicating that they are a unique combination associated with the ASD status in this population. The findings also demonstrate that about 50% of ASD children do not fail these milestones during the second year of life. Thus, ASD-specific screening is needed at later ages to detect children without early developmental concerns.

Some milestones from the second year of life which appear in early screeners for ASD did not enter the model including eye contact, response to name, and expressing needs. It is possible that these markers had a weaker signal since they require closer clinical observation than a brief surveillance checkup and/or warrant a quality measuring scale rather than a pass or fail mark. Further improvement of the developmental protocol to target ASD-specific markers is warranted.

Implementing the model with subgroups of the population yielded relatively stable results. For the boys-only model, 10 of the consistently important features overlapped with the consistent ones in the full-model. Birthweight was only a consistent feature across the boys-only model, supporting evidence for differences in birth parameters of boys versus girls with ASD [28]. Since most of the ASD sample comprised males, there is need to replicate the model with a large sample of girls to detect different early “red flags”.

There were strikingly higher rates of ASD among mothers born in Russia and, to some degree, among those from Ethiopian origins relative to their proportion in the population (see Table 1). Ethnicity and socioeconomic status are inconsistently linked with lower rates of ASD [4]. In contrast with our findings, lower rates of ASD were reported in lower socioeconomic status (SES) groups [3] and, specifically, in non-native Israeli families, including Ethiopian ones [56]. Previous research differs dramatically in terms of ascertainment and publication years. Higher rates of early ASD diagnosis in immigrant communities may relate to a combination of biological risk factors, increased discrimination, isolation and stress levels, and poor prenatal care, as well as provider biases in immigrant groups. Future research into the healthcare and child contributing factors is critical to ensure equality in early detection.

While our study does not explicitly focus on the disparities between developed and developing countries in the detection of ASD, it is crucial to consider how such a model would perform in diverse healthcare contexts. Though our research does not delve into the specifics of this issue, we have included data that explore the role of immigration in mothers on their children’s ASD detection rates. Previous findings using the Modified Checklist for Autism in Toddlers (MCHAT) in a pediatric healthcare setting also showed higher rates of positive screenings among children of color and those from lower-income households compared with white, higher-income, privately insured, and suburban children [57]. As opposed to a specific tool, designing a screening system that relies on routinely collected data enables ASD detection that is based upon multi-cultural representative data. Carrying over such technology to developing countries requires direct cross-cultural validation at multiple levels, including the expression of signs, parental recognition, interpretation, and reporting levels [58].

## 5. Limitations

Integrating developmental milestones in the model required accounting for the variability in timing of checkups as well as multiple entries per milestone per child. To avoid attributing a-priori developmental assumptions, for instance to: ‘never tested’ and ‘late tested’ data, milestones were transformed to binary features of ‘ever passed’ and ‘only failed’. While this enabled us to minimize the attribution of late performance to later development, we were not able to account for children who passed after several attempts. Future ML work that integrates a child’s pattern of performance over time may improve the model’s capacity to capture delay.

The ML model is limited to the list of milestones and risk factors examined and recorded in national surveillance check-ups. Additional early signs linked to subsequent ASD diagnosis were not examined, and as such, were not identified in our study. Milestones such as head lag at 3 months, raising hands to be picked up at 6 months, and nodding at 12 months are not included in the formal examination and may potentially be important “red flags” that strengthen the model [59]. In addition, father age and family history of ASD are two known risk factors [51] that are not routinely collected and would be important to add in future research for promoting better ASD detection. Advanced maternal age was found to be significant, and it may be a proxy for advanced paternal age.

An additional limitation is that our identification of ASD is based on reports during visits. It is plausible that there is a much larger ASD group that has been diagnosed later or not reported during well-baby visits. Based on the national incidence of ASD, the true positive ASD group is at least 10 times higher in the examined population [5]. Lower rates of ASD also relate to the young age of this community sample (born 2014–2019). However, as the model relied on accurate and early ASD diagnoses, it enhances its reliability in identifying the group that has not been previously identified in the EHR through earlier detection in future assessments. We attempted to identify some of these children through the estimation of global developmental delay among the high-risk group. This adds a lot of noise to the outcome label, and thus, requires replication with a later stable outcome label. In addition, there was variability in the way providers recorded diagnosis, with 43.37% of the ASD diagnoses ascertained via provider notes rather than ICD-9 code. Further research into inter-rater assessment differences in clinics is warranted.

## 6. Conclusions

This study highlights the value of ML systems for detecting elevated ASD signs in preventative care. The most important predicting features in our models reflected a mixture of biometric and clinical data. The representative nature of this rich database enables us to account for the medical and behavioral heterogeneity of young children. Although clinical data are noisy, the model showed high accuracy levels, identifying most children with ASD prior to 2 years of age. While the model had a cost of high false positive rates, some of these children showed other developmental issues that required verification. The model pointed to significant milestones and features that are easy to recognize and can be incorporated in detecting children that mandate a more thorough and systematic ASD-focused evaluation. Future work is needed to integrate such data-driven ML systems to support providers in personalizing early detection of ASD during routine developmental checkups.

## Figures and Tables

**Table 1:** Comparison of child and family demographic between groups.

Characteristics	TD (*N* = 774,778)	ASD (*N* = 1163)	Statistics
Year of child’s birth			χ^2^(5) = 106.49 ***, φ_c_ = 0.012
2014	124,401 (16.1%) _a_	174 (15%) _a_	
2015	126,679 (16.4%) _a_	230 (19.8%) _b_	
2016	129,421 (16.7) _a_	278 (23.9%) _b_	
2017	131,721 (17%) _a_	212 (18.2%) _a_	
2018	132,522 (17.1%) _a_	182 (15.6%) _a_	
2019	130,034 (16.8%) _a_	87 (7.5%) _b_	
Mother birth country			χ^2^(5) = 375.48 ***, φ_c_ = 0.023
Israel	631,588 (81.5%) _a_	816 (70.2%) _b_	
Russia	43,766 (5.6%) _a_	209 (18%) _b_	
Europe	1750 (1.6%) _a_	6 (0.5%) _b_	
Ethiopia	11,114 (1.4%) _a_	40 (3.4%) _b_	
North America	8799 (1.1%) _a_	3 (0.3%) _b_	
Other	8360 (1.3%) _a_	18 (1.5%) _a_	
Missing	58,401 (7.5%)	71 (6.1%)	
Mother Employment Status			χ^2^(2) = 11.34, φ_c_ = 0.005
Employed	334,375 (43.2%) _a_	512 (44%) _a_	
Unemployed	164,258 (21.2%) _a_	271 (23.3%) _a_	
Student	32,129 (4.1%) _a_	27 (2.3%) _b_	
Missing	244,016 (31.5%)	353 (30.4%)	

Note. Continuous variables were compared using independent samples *t*-test, while nominal variables with Chi square or Fisher exact tests. Missing data were not included in the comparisons. ASD: Autism spectrum disorder. TD: Typical development. _a,b_ Groups with different subscripts are significantly different based on pairwise adjusted Z-tests. *** *p* < 0.001.

**Table 2:** Comparison of familial and birth parameters between groups.

Characteristics	TD (*N* = 774,778)	ASD (*N* = 1163)	Statistics
Mother’s age _c_ Mean (SD, Min–Max)	30.02 (5.71, 15–55)	31.43 (5.65, 19–52)	t (767,831) = −8.27 ***, d = 0.24
Missing	8688 (1.12%)	2 (0.17%)	
Birth weight (Kg) Mean (SD, Min–Max)	3.21 (0.51, 0.4–6)	3.15 (0.58, 0.72–4.77)	t (747,034) = 3.96 ***, d = 0.1
Missing	28,871 (3.72%)	34 (2.92%)	
Pregnancy week _c_ Mean (SD, Min–Max)	39.09 (1.8, 23–43)	38.65 (2.07, 25.2–42.3)	U = 437,787,198 ***, d = 0.004
Missing	29,465 (3.8%)	32 (2.75%)	
Males _c_ N (%)	398,069 (51.4%)	911 (78.3%)	***, φ = 0.021
Preterm births (pregnancy week < 37)	54,937 (7.1%)	132 (11.3%)	***, φ = 0.006
Missing	41,496 (5.4%)	52 (4.5%)	
Birth type			χ^2^(2) = 86.04 ***, φ_c_ = 0.011
Spontaneous	539,004 (69.6%) _a_	693 (59.6%) _b_	
Cesarean	129,685 (16.7%) _a_	309 (26.6%) _b_	
Instrumental	37,232 (4.8%) _a_	71 (6.1%) _b_	
Missing	68,857 (8.9%)	90 (7.7%)	
Multiple Pregnancy			χ^2^(3) = 18.87 ***, φ_c_ = 0.005
Single	741,871 (95.8%) _a_	1084 (93.2%) _b_	
Twin	31,783 (4.1%) _a_	76 (6.5%) _a_	
Triplet	1058 (0.1%) _a_	3 (0.3%) _a_	
Other	66 (0.001%) _a_	0 (0%) _a_	

Note. Continuous variables were compared using independent samples *t*-test, while nominal variables with Chi square or Fisher exact tests. Missing data were not included in the comparisons. ASD: Autism spectrum disorder. TD: Typical development. _a,b_ Groups with different subscripts are significantly different based on pairwise adjusted Z-tests. _c_ These features were among the top important features in the final model. *** *p* < 0.001.

**Table 3:** Comparison of post-natal parameters between groups.

Characteristics	TD (*N* = 774,778)	ASD (*N* = 1163)	Statistics
Postpartum depression			χ^2^(3) = 7.19, φ_c_ = 0.004
None	539,565 (69.6%) _a_	817 (70.2%) _a_	
Mild	11,941 (1.5%) _a_	16 (1.4%) _a_	
Moderate	2306 (0.3%) _a_	8 (0.7%) _b_	
Severe	1062 (0.1%) _a_	3 (0.3%) _a_	
Missing	219,904 (28.3%)	319 (27.4%)	
Ever had Concern for Development _c_	71,516 (9.2%)	820 (70.5%)	***, φ = 0.08
Missing	10,162 (1.3%)	8 (0.7%)	
Ever had Hearing Suspicion	22,319 (2.9%)	272 (23.4%)	***, φ = 0.047
Missing	16,200 (2.1%)	12 (1%)	
Ever Nursing _c_	584,345 (75.4%)	708 (60.9%)	***, φ = −0.014
Missing	11,830 (1.5%)	13 (1.1%)	
Ever had Anemia	41,589 (5.4%)	58 (5%)	
Missing	388 (0.1%)	0 (0%)	

Note. Continuous variables were compared using independent samples *t*-test, while nominal variables with Chi square or Fisher exact tests. Missing data were not included in the comparisons. TD: Typical development. ASD: Autism spectrum disorder _a,b_ Groups with different subscripts are significantly different based on pairwise adjusted Z-tests. _c_ These features were among the top important features in the final model. *** *p* < 0.001.

**Table 4:** Comparison of 3-fold cross-validation using different methods.

Method	Average AUC	SD	TP	FP	TN	FN	Sensitivity	Specificity
Logistic regression	0.86	0.010	897	159,472	615,305	266	0.77	0.79
Random Forest	0.84	0.009	861	166,793	607,984	302	0.74	0.78
Naive bayes	0.83	0.011	888	185,027	589,750	275	0.76	0.76

## Data Availability

The data presented in this study are available on request from the TIMNA-Israel Ministry of Health’s Big Data Platform, Ministry of Health and are available at https://govextra.gov.il/ministry-of-health/big-data-research/home/ (accessed on 31 December 2021), Jerusalem, Israel. The data are not publicly available due to privacy and ethical restrictions.

## Supplementary Materials

The following supporting information can be downloaded at: https://www.mdpi.com/article/10.3390/children11040429/s1, Table S1: List of 100 features; Table S2: Model performance for training data; Table S3: Comparison of model performance with different thresholds (various multiples of the Youden Threshold); Table S4: Accuracy measures of the Full Model, Boys Model, and No-2019 Model; Figure S1: Feature importance bar plot and SHAP summary plot for a fold 1 from the Full Model; Figure S2: Feature importance bar plot and SHAP summary plot for a fold 3 from the Full Model; Figure S3: Feature importance bar plot and SHAP summary plot for a fold 1 from the Boys Model; Figure S4: Feature importance bar plot and SHAP summary plot for a fold from the Boys Model; Figure S5: Feature importance bar plot and SHAP summary plot for fold 3 from the Boys Model.
