# Supplementary material for: A Prediction Model of Autism Spectrum Diagnosis from Well-Baby Electronic Data Using Machine Learning
Source: Children (Basel). 2024 Apr 3;11(4):429. doi: 10.3390/children11040429 (PMC11049145; doi:10.3390/children11040429)
Supplement: Supplementary file 1 [file children-11-00429-s001.zip › children-2936354-supplementary.pdf]

Supplemental materials

Supplemental material Table S1. List of 100 Features.

Category	Feature
Familial	Multiple pregnancy[d]
	Mother Age[a]
Birth parameters	Pregnancy Week[ae]
	Sex[a]
	Birth Weight[c]
	Birth Length[d]
	Birth Type Unknown[d]
	Birth Type instrumental[d]
	Birth Type Cesarean[b]
	Birth Type Spontaneous[d]
	Prematurity[d]
Post-natal	Nursing[a]
	Anemia[d]
	Depression Questionnaire Score[d]
	Parent Concern for Hearing Loss
	Parent Concern for Development[a]
Growth	Weight by Height %ile SD[a]
	Weight by Height %ile Median[c]
	First Weight by Height %ile[a]
	Last Weight by Height %ile[a]
	Head Circumference %ile SD[b]
	Head Circumference %ile Median[b]
	First Head Circumference %ile[a]
	Last Head Circumference %ile [b]
Developmental progress	Visually follows a moving object horizontally- Passed
	Visually follows a moving object horizontally- Failed[d]

	Vocalizes in response to human voice- Passed
	Vocalizes in response to human voice- Failed[d]
	Smiles responsively- Passed
	Smiles responsively- Failed[d]
	Raises head- Passed
	Raises head- Failed[d]
	Hands together, manipulates fingers- Passed
	Hands together, manipulates fingers- Failed[d]
	Grasps an object- Passed
	Grasps an object- Failed[d]
	Visually follows a moving object vertically- Passed[d]
	Visually follows a moving object vertically- Failed[d]
	Responds to rattling sound- Passed
	Responds to rattling sound- Failed[d]
	Makes various sounds including constants (ie, Mm rr gg) – Passed[b]
	Makes various sounds including constants (ie, Mm rr gg) – Failed[d]
	Responds to human presence-Passed
	Responds to human presence- Failed[d]
	Head and chest up in prone position – Passed
	Head and chest up in prone position- Failed[d]
	Rolls over from abdomen to back and back to abdomen- Passed
	Rolls over from abdomen to back and back to abdomen- Failed
	Crawls- Passed
	Crawls- Failed
	Transfers an object from one hand to the other- Passed
	Transfers an object from one hand to the other- Failed[d]
	Taps 2 objects playfully- Passed
	Taps 2 objects playfully- Failed
	Makes repetitive syllables- constant or vowels- Passed
	Makes repetitive syllables- constant or vowels- Failed

	Gets to sit without support- Passed
	Gets to sit without support- Failed
	Pulls to stand- Passed
	Pulls to stand- Failed[d]
	Uses thumb- finger grasp- Passed
	Uses thumb- finger grasp- Failed[d]
	Feeds self- Passed
	Feeds self- Failed[d]
	Vocalizes in a dialogue- Passed
	Vocalizes in a dialogue- Failed[d]
	Understands simple instructions- Passed
	Understands simple instructions- Failed[d]
	Responds when addressed by name- Passed
	Responds when addressed by name- Failed[d]
	Says one word or pronounces meaningful sounds- Passed
	Says one word or pronounces meaningful sounds- Failed[d]
	Responds differently to familiar and stranger- Passed
	Responds differently to familiar and stranger- Failed[d]
	Walks with assistance- Passed
	Walks with assistance- Failed[d]
	Says 2-3 words- Passed[d]
	Says 2-3 words- Failed[c]
	Familiar with at least 1 body part- Passed[a]
	Familiar with at least 1 body part- Failed[d]
	Points at familiar objects to request- Passed
	Points at familiar objects to request- Failed[b]
	Express will vocally or with gestures- Passed
	Express will vocally or with gestures- Failed[d]
	Makes eye contact and express reciprocity during joint game- Passed
	Makes eye contact and express reciprocity during joint game- Failed[d]

	Walks without assistance- Passed
	Walks without assistance- Failed
	Climbs upstairs with assistance- Passed
	Climbs upstairs with assistance- Failed
	Builds a tower of cubes- Passed
	Builds a tower of cubes- Failed[a]
	Eats independently with a spoon- Passed
	Eats independently with a spoon- Failed[a]
	Has a vocabulary of over ten words- Passed
	Has a vocabulary of over ten words- Failed[a]
	Composes a sentence of at least two word- Passed[a]
	Composes a sentence of at least two word- Failed[d]
	Squeezes and sticks out lips to give a kiss- Passed
	Squeezes and sticks out lips to give a kiss- Failed[a]

Note. [a]Among the 15 features that consistently appeared in the top 20 important features across all 3 folds of the full model. [b]Among the 6 features that consistently appeared in the top 20 important features across 2 of the 3 folds of the full model.

[c]Among the 3 features that consistently appeared in the top 20 important features across 1 of the 3 folds of the full model.

[d]Among the 35 features that were entered into the model but were not in the 20 important features of any of the folds. [e]Pregnancy week and prematurity were counted as reflecting one conceptually similar feature.

Supplemental material Table S2. Model Performance for Training Data**.**

	AUC	Sensitivity	Specificity
Full model folds:			
1	0.88	0.76	0.83
2	0.87	0.79	0.79
3	0.88	0.78	0.82
Boys model folds:			
1	0.85	0.72	0.83
2	0.86	0.71	0.84
3	0.83	0.72	0.80

Supplemental material Table S3. Comparison of Model Performance with Different Thresholds (various multiples of the Youden Threshold)**.**

Multiplication Value of Youden Threshold	TP	FP	TN	FN	Sensitivity	Specificity	PPV	NPV
0.25	1117	529153	245624	46	0.96	0.32	0.00	1.00
0. 5	1001	290813	483964	162	0.86	0.62	0.00	1.00
2	724	79141	695636	439	0.62	0.90	0.01	1.00
4	551	35583	739194	612	0.47	0.95	0.02	1.00

Note. AUC: Area under the ROC curve. TP: True positive. FP: False positive. TN: True negative. FN: False negative. Higher multiplication values yield lower FP rates at the cost of lower TP rates.

Supplemental material Figure S1. Feature Importance Bar Plot and SHAP Summary Plot For a fold 1 from the Full Model.

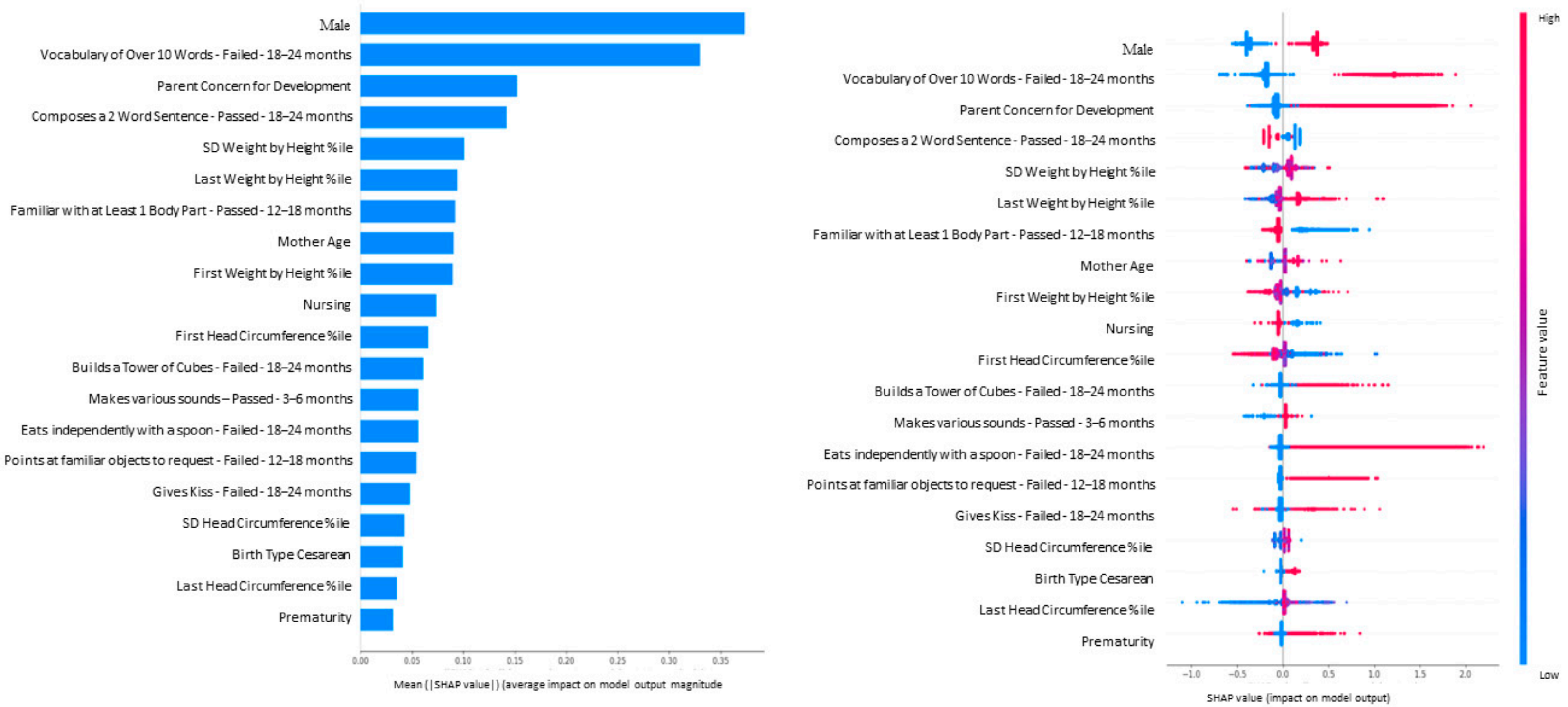

[a] Feature importance bar plot

[b] SHAP summary plot

Supplemental material Figure S2. Feature Importance Bar Plot and SHAP Summary Plot For a fold 3 from the Full Model.

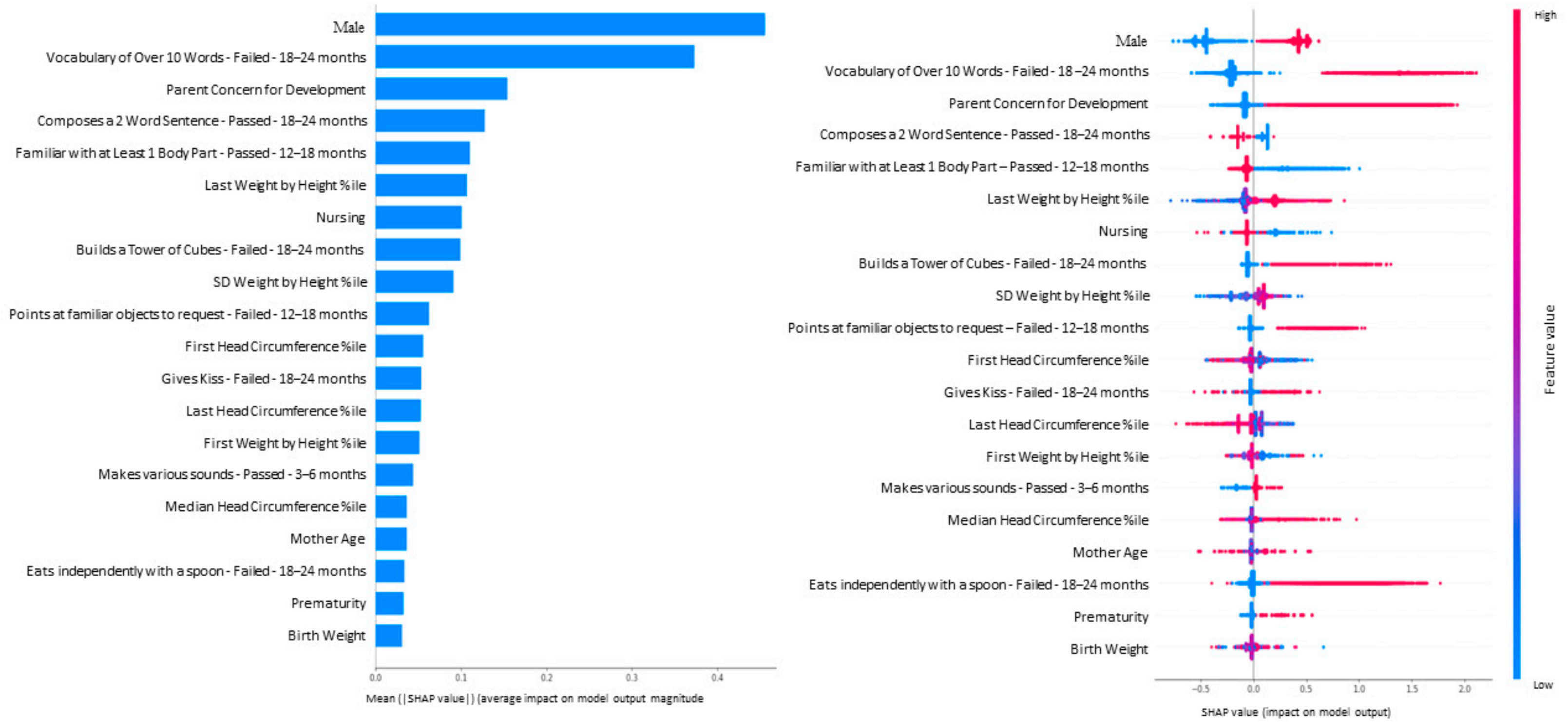

[a] Feature importance bar plot

[b] SHAP summary plot

Note. Fold 2 is presented in the manuscript.

Supplemental material Figure S3. Feature Importance Bar Plot and SHAP Summary Plot For a fold 1 from the Boys Model.

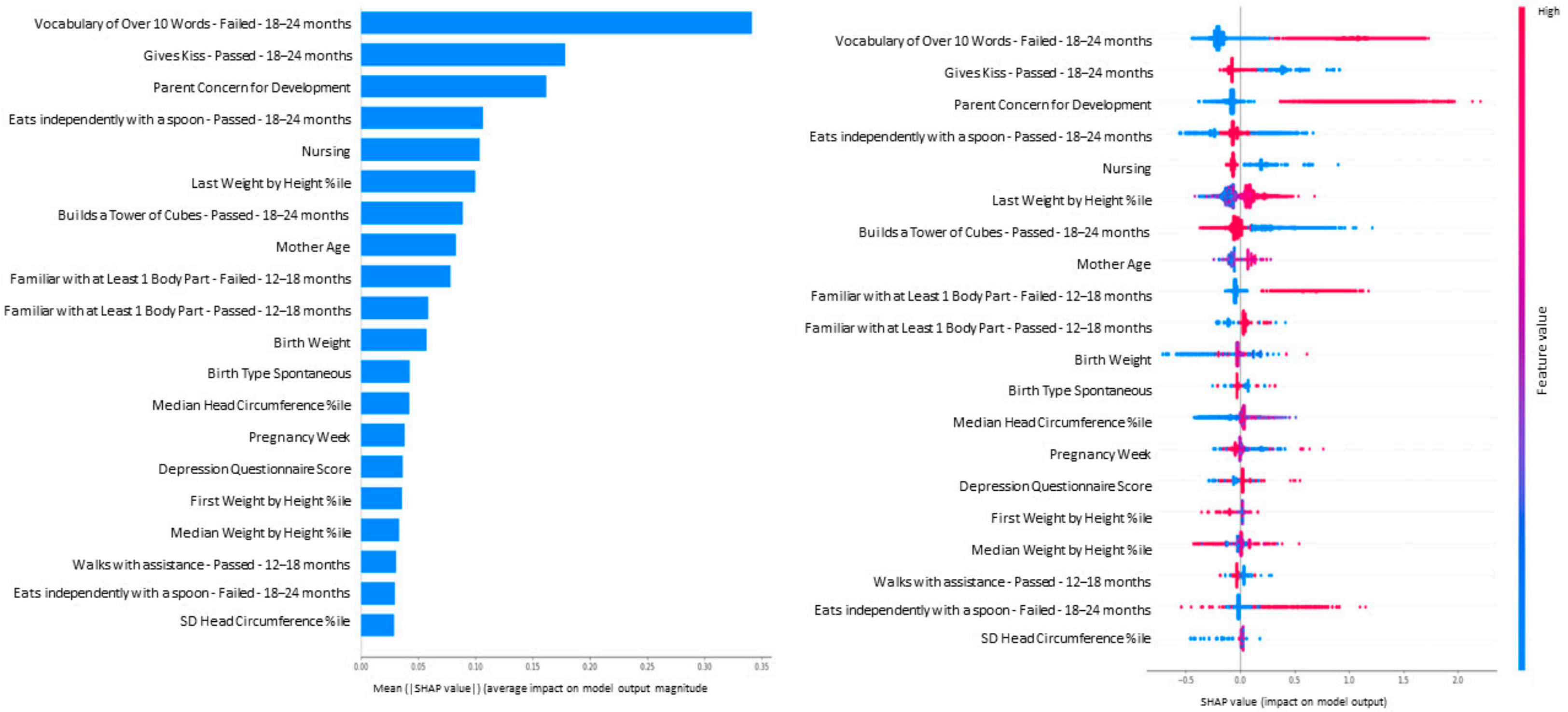

[a] Feature importance bar plot

[b] SHAP summary plot

Supplemental material Figure S4. Feature Importance Bar Plot and SHAP Summary Plot For a fold from the Boys Model.

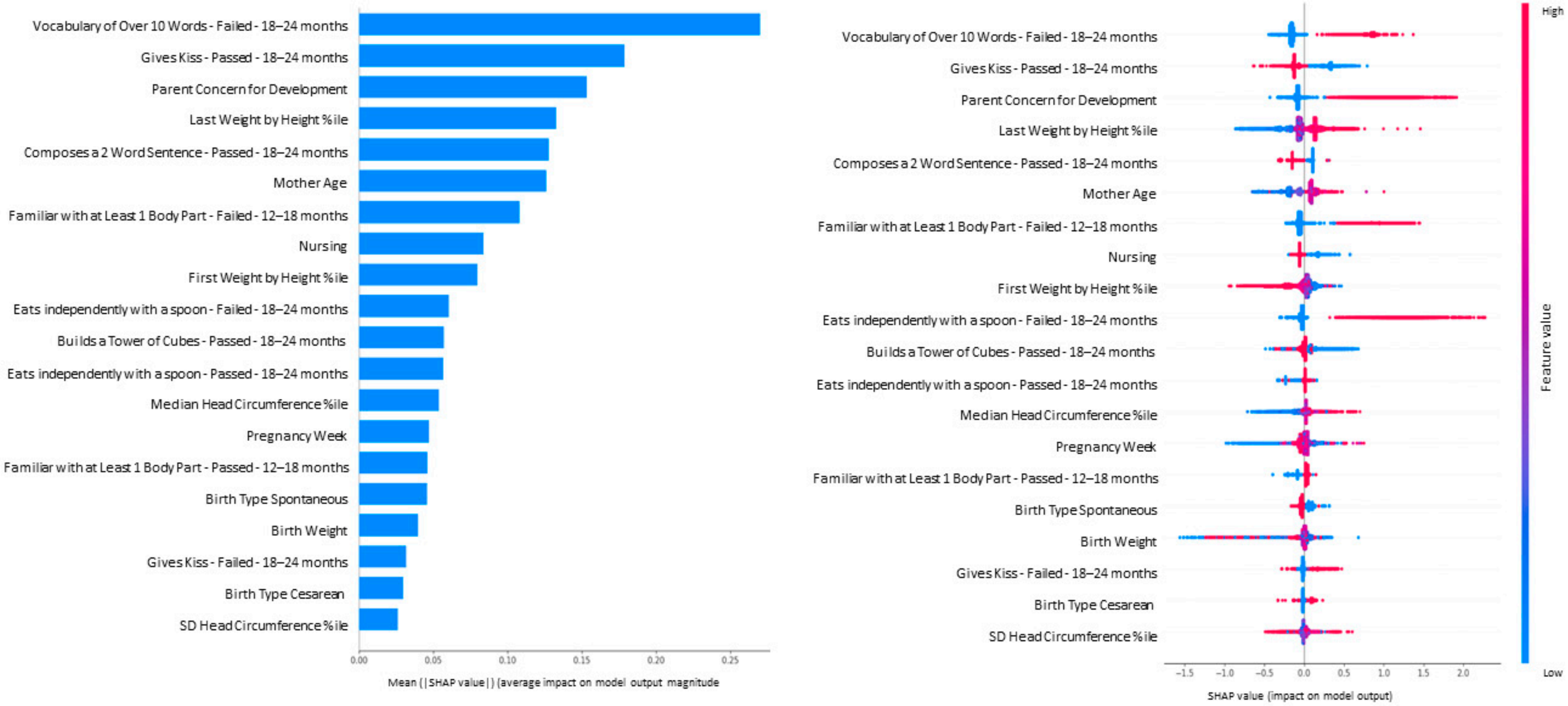

[a] Feature importance bar plot

[b] SHAP summary plot

Supplemental material Figure S5. Feature Importance Bar Plot and SHAP Summary Plot For fold 3 from the Boys Model.

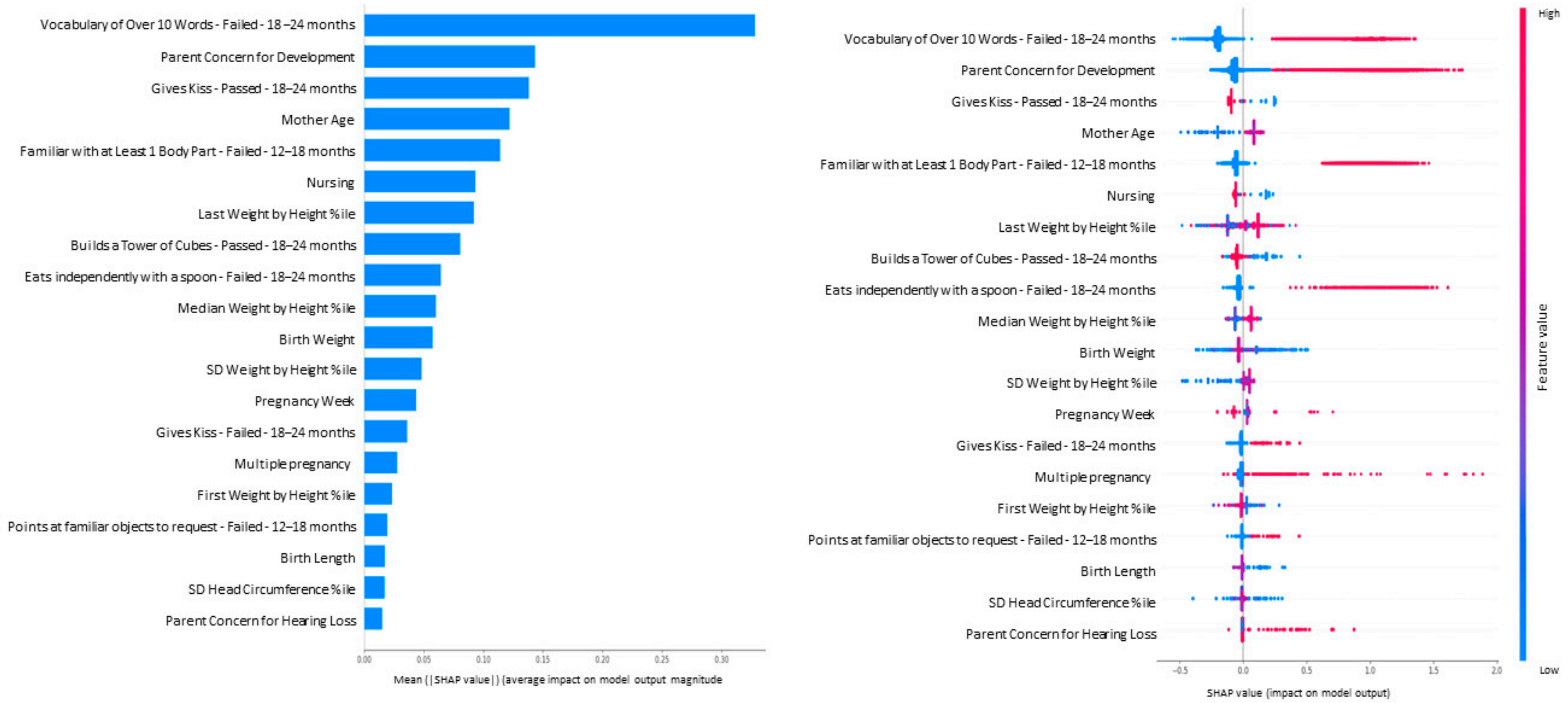

a Feature importance bar

b SHAP summary plot

Supplemental material Table S4. Accuracy Measures of the Full Model, Boys Model, and No-2019 Model.

	Average AUC	SD	TP	FP	TN	FN	Sensitivity	Specificity	PPV	NPV
Full model	0.86	0.01	874	143758	631019	290	0.75	0.81	0.01	1.00
Boys model	0.82	0.03	621	70621	327447	290	0.68	0.82	0.01	1.00
No 2019 model	0.85	0.01	792	118915	525828	284	0.74	0.82	0.01	1.00

Note. AUC: Area under the ROC curve. TP: True positive. FP: False positive. TN: True negative. FN: False negative.
